# Distribution of Major Pilin Subunit Genes Among Atypical Enteropathogenic *Escherichia coli* and Influence of Growth Media on Expression of the *ecp* Operon

**DOI:** 10.3389/fmicb.2018.00942

**Published:** 2018-05-15

**Authors:** Danielle D. Munhoz, Júlia M. Nara, Natália C. Freitas, Claudia T. P. Moraes, Kamila O. Nunes, Bruno B. Yamamoto, Francielli M. Vasconcellos, Ygnacio Martínez-Laguna, Jorge A. Girón, Fernando H. Martins, Cecilia M. Abe, Waldir P. Elias, Roxane M. F. Piazza

**Affiliations:** ^1^Laboratório de Bacteriologia, Instituto Butantan, São Paulo, Brazil; ^2^Centro de Detección Biomolecular, Benemérita Universidad Autónoma de Puebla, Puebla, Mexico

**Keywords:** diarrhea, atypical EPEC, adhesion, fimbriae, ECP

## Abstract

Atypical enteropathogenic *Escherichia coli* (aEPEC) strains are unable to produce the bundle-forming pilus (BFP), which is responsible for the localized adherence pattern, a characteristic of the pathogenicity of typical EPEC strains. The lack of BFP in aEPEC strains suggests that other fimbrial or non-fimbrial adhesins are involved in their adhesion to the host cells. The aim of this study was to investigate the distribution of major subunit fimbrial genes known to be important adherence factors produced by several *E. coli* pathotypes in a collection of 72 aEPEC strains. Our results demonstrate that a high percentage (94–100%) of aEPEC strains harbored *ecpA, fimA, hcpA*, and *lpfA* fimbrial genes. Other fimbrial genes including *pilS, pilV, sfpA, daaC, papA*, and *sfa* were detected at lower frequencies (1–8%). Genes encoding fimbrial subunits, which are characteristic of enteroaggregative *E. coli* or enterotoxigenic *E. coli* were not found. No correlation was found between fimbrial gene profiles and adherence phenotypes. Since all aEPEC strains contained *ecpA*, the major pilin gene of the *E. coli* common pilus (ECP), a subset of *ecpA*+ strains was analyzed for transcription of *ecpRABCDE* and production of ECP upon growth in three different culture conditions at 37°C. Transcription of *ecpRABCDE* occurred in all conditions; however, ECP production was medium dependent. In all, the data suggest that aEPEC strains are highly heterogeneous in terms of their fimbrial gene profiles. Despite lacking BFP production, other mechanisms of cell adherence exist in aEPEC strains to ensure host colonization, e.g., mediated by other prevalent pili such as ECP. Moreover, the production of ECP by aEPEC strains might be influenced by yet unknown post-transcriptional factors.

## Introduction

Bacterial adherence to host tissues is a multifactorial process involving distinct fimbrial and non-fimbrial adhesins that act in concert at different stages during infection. So far, a variety of fimbrial types has been identified and characterized in bacteria (Ofek et al., 2003; Jonson et al., 2005). The chromosomes of commensal and pathogenic *Escherichia coli* typically harbor between 12 and 16 different pili operons but the function of the majority of these pili systems and whether they are expressed or not remain uncharacterized (Brunder et al., 2001; Doughty et al., 2002; Torres et al., 2002, 2004; Rendón et al., 2007; Xicohtencatl-Cortes et al., 2007; Saldaña et al., 2009b; Samadder et al., 2009; Ross et al., 2015). Epidemiological data gathered around the world indicate that some pili types define some of the different classes of pathogenic *E. coli* and are associated with their adhesive potential and virulence. For example, the plasmid-encoded bundle-forming pilus (BFP), colonization factors (CFs), and aggregative adherence fimbriae (AAF) are found in enteropathogenic (EPEC), enterotoxigenic (ETEC), and enteroaggregative (EAEC) *E. coli* strains, respectively (Girón et al., 1991; Nataro et al., 1992; Czeczulin et al., 1997; Bernier et al., 2002; Qadri et al., 2005; Boisen et al., 2008; Jønsson et al., 2015). Type 1 pili, the long polar fimbriae (LPF), and the *E. coli* common pilus (ECP) are among the ubiquitous fimbrial adhesins of *E. coli* pathotypes. The ECP that has been detected in enterohemorrhagic *E. coli* (EHEC), EPEC, EAEC, ETEC, uropathogenic and avian pathogenic *E. coli* (Rendón et al., 2007; Blackburn et al., 2009; Saldaña et al., 2009a, 2014; Avelino et al., 2010; Hernandes et al., 2011; Stacy et al., 2014). In typical EPEC (tEPEC) strains, ECP appears to act synergistically with BFP during formation of the localized adhesion (LA) pattern (Saldaña et al., 2009a).

A subclass of EPEC strains lacking BFP genes has emerged in several regions of the world as an important cause of childhood diarrhea (reviewed in Gomes et al., 2016). This subclass is referred to as atypical EPEC (aEPEC) and does not produce the typical LA on cultured epithelial cells associated with BFP production (Scaletsky et al., 1984). Instead, aEPEC strains adhere poorly to cultured cells forming loose clusters, a pattern called localized-adherence like (LAL) (Rodrigues et al., 1996; Scaletsky et al., 1999). Some studies have demonstrated that aEPEC strains harbor a wide range of fimbrial genes in different combinations (Gomes et al., 2004; Afset et al., 2006; Tennant et al., 2009; Scaletsky et al., 2010; Hernandes et al., 2011; Piazza et al., 2013). However, only a few studies have characterized the expression of fimbrial genes in aEPEC strains (Hernandes et al., 2011; Nascimento et al., 2014). Thus, it is important to search for highly prevalent virulence markers in this pathotype in order to better understand their adherence mechanisms aiming to identify targets for diagnosis and/or prevention of aEPEC infections.

Therefore, the aim of the present study was to investigate the distribution of known pathogenic *E. coli* fimbrial adhesins genes in aEPEC strains displaying different adherence phenotypes. Since the *ecpA* gene was present in all the aEPEC strains tested, we also investigated the influence of growth media in the differential expression and production of ECP among these strains to gain knowledge on the regulation of ECP.

## Materials and Methods

### Bacterial Strains

We studied all 72 aEPEC strains isolated from children with diarrhea in a case-control survey conducted between 2003 and 2004 in the city of Salvador, Brazil (Bueris et al., 2007). These strains were previously characterized as aEPEC showing the following features: *eae^+^*/EAF^-^/*stx^-^/*BFP^-^ (Abe et al., 2009; Nara et al., 2010). They belong to a wide range of serotypes, and exhibit distinct patterns of adherence: LAL, diffuse adherence (DA), aggregative adherence (AA), non-characteristic, and non-adherent (Abe et al., 2009). Strains used as controls in different assays are listed in Supplementary Table S1.

### Detection of Fimbrial Genes

The following fimbriae-encoding genes were searched among the 72 aEPEC strains by PCR: *fimA, fimH, papA, sfaD-E, bfpA, ecpA, espA, ldaH, aggA, aafA, agg3A, agg4A, pilS, pilV, lngA, cfaB, cooA, cotA, cstA, cofA, csaA, csfA, cssA, daaC, sfpA, hcpA, lpfA_O113_, lpfA1-1, lpfA1-2, lpfA1-3, lpfA1-5, lpfA2-1*. Primer sequences, annealing temperatures and sizes of amplified fragments are listed in Supplementary Table S1. Amplification was performed in a total volume of 50 μL containing: dATP, dTTP, dCTP, and dGTP (0.1 mM each), 1.5 U *Taq* DNA polymerase, 5.0 μL 10x PCR buffer and 2 mM MgCl_2_ (Invitrogen, Boston, MA, United States), 40 pmol of each primer, and 2.0 μL of DNA template, obtained from a colony from culture on Luria-Bertani (LB) agar boiled in 300 μL of water for 10 min. The PCR was carried out at 94°C for 5 min, 30 cycles of 94°C for 1 min, annealing temperature (see Supplementary Table S1 for specific temperatures and incubation times), 72°C for 1 min, followed by a final extension for 5 min at 72°C. The PCR products were analyzed by agarose gel (0.7–1%) electrophoresis after GelRed staining (Uniscience, Miami, FL, United States), and the images captured by the AlphaImage^TM^ 2200 program (Alpha Innotech, San Leandro, CA, United States). Twenty-two LAL aEPEC strains were selected for PCR analysis of *ecpRABCDE* genes as described above.

### Growth Conditions for Analysis of ECP Production

Among the 72 aEPEC strains tested, four LAL-producing aEPEC (BA2103, BA3378, BA4132, and BA4147) were selected for ECP production. For analysis of *ecpRABCDE* transcription and ECP production the strains were initially grown in LB at 37°C for 18 h. The bacterial cultures were then transferred to LB, Dulbecco’s modified Eagle Medium (DMEM) or preconditioned DMEM (PC-DMEM) (1:100) followed by static incubation at 37°C until a final absorbance of 0.6 at 600 nm (A_600_). PC-DMEM was prepared by incubating DMEM (without antibiotics and fetal bovine serum) in the presence of monolayers of HeLa cells for 48 h at 37°C. The supernatant referred to as “preconditioned medium” was collected, adjusted to pH 7.4, and filtered through a 0.2 μm pore membrane (Girón et al., 2002). tEPEC E2348/69 and *E. albertii* 1551-2 strains (Levine et al., 1985; Yamamoto et al., 2017) were used as positive and negative controls of ECP production, respectively.

### Transcription of *ecp* Operon

Strains BA2103, BA3378, BA4132 and BA4147, as well as appropriate control strains were grown in LB, DMEM and PC–DMEM and centrifuged at 4,000 × *g* for 5 min. Pellets were resuspended in 1 mL of the respective culture media and subsequently used for RNA extraction using RNA Protect and RNeasy Mini kit (Qiagen, Hilden, Germany), according to the manufacturer’s recommendations. cDNAs were synthesized using 1 μg of RNA for each strain, by Superscript III First-Strand Synthesis Systems kit for RT-PCR (Invitrogen, Boston, MA, United States), following the manufacturer’s recommendations. The presence of *ecpRABCDE* genes in the cDNAs obtained were assessed by PCR as described in **Table 1**. PCR for the constitutive gene *rpoA* was included as control of cDNA integrity. All reactions were performed in triplicates in three independent experiments.

**Table 1:** Primer sequences of *ecp* operon and PCR conditions used to study *ecp* operon.

Gene	Sequence	Annealing temperature (°C)	Mg (mM)	Fragment (bp)	Reference
*ecpA*	(F) ACCTCGGGAAGAAAAGCAA	56	2	640	This study^∗^
	(R) CAATTCCGTCCAGGAATAAA				
*ecpB*	(F) TCTGATGTACCAGCAGGG	47	1.5	780	This study^∗^
	(R) CTTTCAGTCCTGGGGAGA				
*ecpC*	(F) ACGACAATGCCTTTACGAG	57	1	1,130	This study^∗^
	(R) CGATCCATATGAAAGCTACG				
*ecpD*	(F) AGTTTGTGTTTGTCGAAAAC	56	1	760	This study^∗^
	(R) GCCGAGGCTAACGACGA				
*ecpE*	(F) CGGTGGATGGTGAACTACTT	60	2	458	This study^∗^
	(R) CGGACAGGAATCCGTTAATCT				
*ecpR*	(F) CTGTAAAAATTATAGGTTTG	57	2	683	This study^∗^
	(R) ACCAGAGCTATTGCCAGA				

*^∗^Primers designed using *ecp* operon sequence of tEPEC E2348/69 (GenBank accession number: NC_011601).*

### Production of ECP

The production of ECP was studied by immunofluorescence microscopy (IFM) in LAL aEPEC strains BA2103, BA3378, BA4132, and BA4147 growing in the three different media described above. Briefly, the 18 h bacterial cultures were centrifuged for 5 min at 900 × *g* and the pellets were resuspended in 10 μL of the respective medium and fixed on glass slides with 4% p-formaldehyde at 4°C for 18 h. The slides were washed twice with 0.01 M phosphate buffer saline pH 7.4 (PBS), blocked with 10% goat serum in PBS (GS–PBS) for 1 h at room temperature, followed by incubation at 4°C for 18 h with rabbit anti-EcpA antibody (diluted 1:1,000 in GS–PBS). The slides were washed twice with PBS and incubated with goat anti-rabbit IgG conjugated with FITC (Sigma-Aldrich, St. Louis, MO, United States) diluted 1:500 in GS–PBS at room temperature for 1 h. After incubation, the slides were washed twice with PBS and mounted with Vectashield antifade mounting medium (Vector Laboratories, Burlingame, CA, United States) and covered with glass coverslips. The preparations were visualized under laser scanning confocal (LSM 510 META, Zeiss, Oberkochen, Germany) microscopes with original magnification of 1,000×. Triplicates of three independent experiments were performed.

We also determined ECP production in cultures of strain BA2103 obtained in LB, DMEM or PC–DMEM by transmission electron microscopy (TEM) using immunogold-labeling method. Strains E2348/69 and 1551-2 were used as controls. Briefly, preparations were blocked with a solution of 0.2% BSA in PBS and then incubated with rabbit anti-EcpA at 1:100 dilution for 2 h at room temperature. Following this period, the preparations were incubated with goat anti-rabbit antibody labeled with 10 nm colloidal gold particles (Sigma-Aldrich, St. Louis, MO, United States) at 1:50 dilution for 2 h at room temperature. Preparations were negatively stained with 2% uranyl acetate in water, applied onto formvar-coated nickel grids and observed under TEM (LEO 906E – Zeiss, Oberkochen, Germany) at 80 kV. Triplicates of three independent experiments were performed.

## Results

### *E. coli* Fimbrial Genes Are Found in aEPEC Strains in Different Combinations

**FIGURE 1:**
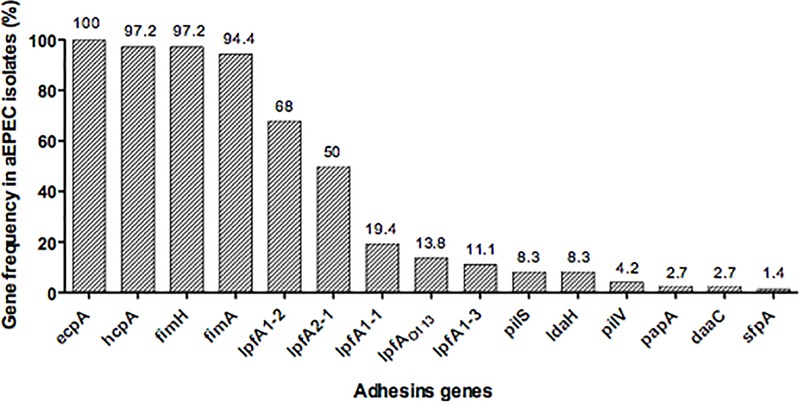
Distribution of the frequency of fimbrial genes among 72 aEPEC strains studied.

Fifteen fimbrial genes were detected in our collection: *ecpA* (100%), *fimH* and *hcpA* (97.2%), *fimA* (94.4%), *lpfA1-2* (68%), *lpfA2-1* (50%), *lpfA1-1* (19.4%), *lpfA_O113_* (13.8%), *lpfA1-3* (11.1%), *pilS* and *ldaH* (8.3%), *pilV* (4.2%), *papA and daaC* (2.7%) and *sfpA* (1.4%) (**Figure 1**). This large repertoire of fimbrial genes was distributed in different combinations among the 72 strains, demonstrating no correlation between fimbrial gene profiles and adherence pattern (**Table 2**). None of the aEPEC strains contained the following fimbrial genes: *aggA, aafA, agg3A, agg4A, cooA, cotA, cstA, cofA, csaA, csfA, cssA, lngA, lpfA1-5*, and *sfa* (**Table 2**).

**Table 2:** Genetic fimbrial profile of aEPEC strains.

Strains	Fimbrial adhesins profile	Adherence pattern	Serotype
BA558	*pilS, pilV, lpfA1-2, lpfA1-3, ecpA, hcpA*	LA	O111:H40
BA1652	*fimA, fimH, ecpA, hcpA*	LAL	O131:H4
BA2853	*fimA, fimH, ecpA, hcpA*	LAL	ONT:H10
BA487	*fimA, lpfA1-3, ecpA, hcpA*	LAL	O55:H7
BA714	*fimH, lpfA1-2, ecpA, hcpA*	LAL	O111:H-
BA2034	*fimA, fimH, lpfA1-2, ecpA, hcpA*	LAL	ONT:H10
BA3733	*fimA, fimH, lpfA1-2, ecpA, hcpA*	LAL	O119:H19
BA4095	*fimA, fimH, lpfA1-1, ecpA, hcpA*	LAL	O4:H45
BA1649	*fimA, fimH, lpfA2-1, ecpA, hcpA*	LAL	O111:H38
BA3378	*fimA, fimH, pilS, ecpA, hcpA*	LAL	O104:H2
BA1250	*fimA, fimH, lpfA1-2, ecpA, hcpA*	LAL	ONT:H6
BA1324	*fimA, fimH, lpfA1-1, lpfA2-1, ecpA, hcpA*	LAL	O34:H45
BA3157	*fimA, fimH, lpfA1-2, lpfA2-1, ecpA, hcpA*	LAL	O119:H2
BA3574	*fimA, fimH, lpfA1-2, lpfA2-1, ecpA, hcpA*	LAL	ONT:H38
BA4047	*fimA, fimH, lpfA1-3, lpfA2-1, ecpA, hcpA*	LAL	O1:H16
BA4077	*fimA, fimH, lpfA1-2, lpfA2-1, ecpA, hcpA*	LAL	O64:H23
BA4147	*fimA, fimH, papA, lpfA1-3, ecpA, hcpA*	LAL	O55:H7
BA320	*fimA, fimH, lpfA1-2, lpfA1-3, ecpA, hcpA*	LAL	O55:H7
BA589	*fimA, fimH, pilS, lpfA1-2, lpfA2-1, ecpA, hcpA*	LAL	O5:H2
BA3851	*fimA, fimH, lpfA1-1, lpfA1-2, lpfA2-1, ecpA, hcpA*	LAL	ONT:H38
BA4132	*fimA, fimH, lpfA1-1, lpfA1-2, lpfA2-1, ecpA, hcpA*	LAL	O51:H48
BA3977	*fimA, fimH, lpfA1-1, lpfA1-2, sfpA, ecpA, hcpA*	LAL	ONT:H45
BA2103	*fimA, fimH, lpfAO113, lpfA1-2, lpfA2-1, ecpA, hcpA*	LAL	O26:H11
BA92	*fimA, fimH, ecpA, hcpA*	AA	O2:H16
BA4157	*fimA, fimH, ecpA, hcpA*	AA	ONT:H25
BA2482	*fimA, fimH, lpfA1-2, ecpA, hcpA*	AA	O119:H11
BA585	*fimA, fimH, lpfA1-2, lpfA1-3, ecpA, hcpA*	AA	O157:H16
BA3690	*fimA, fimH, lpfA1-2, lpfA2-1, ecpA, hcpA*	AA	O111:H38
BA2145	*fimA, fimH, lpfAO113, lpfA1-2, lpfA2-1, ecpA, hcpA*	AA	O105:H7
BA2297	*fimA, fimH, lpfA_O113_, lpfA2-1, ecpA*	DA	O153:H11
BA2073	*fimA, fimH, lpfA1-1, lpfA1-2, ecpA*	DA	ONT:H5
BA4009	*fimA, fimH, lpfA_O113_, lpfA1-1, lpfA2-1, ecpA*	DA	O114:H25
BA3170	*fimA, fimH, ecpA, hcpA*	UND	O145:H2
BA4182	*fimA, fimH, ecpA*	UND	O125:H6
BA462	*fimA, fimH, ldaH, ecpA, hcpA*	UND	O51:H40
BA2964	*fimA, fimH, lpfA1-2, ecpA, hcpA*	UND	O51:H40
BA4192	*fimA, fimH, lpfA1-2, ecpA, hcpA*	UND	O111:H25
BA1768	*fimA, fimH, lpfA2-1, ecpA, hcpA*	UND	O51:H40
BA1887	*fimA, fimH, lpfA2-1, ecpA, hcpA*	UND	O111:H38
BA1444	*fimA, fimH, lpfA1-2, lpfA2-1, ecpA, hcpA*	UND	O115:H8
BA2062	*fimA, fimH, lpfA1-2, lpfA2-1, ecpA, hcpA*	UND	O171:H48
BA2459	*fimA, fimH, lpfAO113, lpfA1-2, lpfA2-1, ldaH, ecpA, hcpA*	UND	O26:H11
BA1244	*fimA, fimH, papA, pilS, pilV, lpfA1-2, lpfA1-3, lpfA2-1, daaC, ecpA, hcpA*	UND	O55:H7
BA4058	*fimH, lpfA1-1, ecpA, hcpA*	NA	O20:H-
BA179	*fimA, fimH, ecpA, hcpA*	NA	O23:H16
BA442	*fimA, fimH, lpfA1-2, ecpA, hcpA*	NA	O35:H19
BA580	*fimA, fimH, lpfA1-2, ecpA, hcpA*	NA	O119:H2
BA2294	*fimA, fimH, lpfA1-1, ecpA, hcpA*	NA	O9:H33
BA2613	*fimA, fimH, lpfA1-2, ecpA, hcpA*	NA	O101:H33
BA3392	*fimA, fimH, lpfA1-2, ldaH, ecpA, hcpA*	NA	O124:H11
BA3800	*fimA, fimH, lpfA1-2, ecpA, hcpA*	NA	ONT:H19
BA852	*fimH, lpfA1-2, lpfA2-1, ecpA, hcpA*	NA	O88:H25
BA356	*fimA, fimH, pilS, lpfA1-1, ecpA, hcpA*	NA	O333:H7
BA365	*fimA, fimH, lpfA1-2, lpfA2-1, ecpA, hcpA*	NA	ONT:H19
BA655	*fimA, fimH, lpfA1-2, lpfA2-1, ecpA, hcpA*	NA	O88:H25
BA2065	*fimA, fimH, lpfA1-1, lpfA1-2, ecpA, hcpA*	NA	ONT:H5
BA2117	*fimA, fimH, lpfA1-1, lpfA1-2, ecpA, hcpA*	NA	ONT:H5
BA2975	*fimA, fimH, lpfA1-2, lpfA2-1, ecpA, hcpA*	NA	O88:H25
BA2991	*fimA, fimH, lpfA1-2, lpfA2-1, ldaH, ecpA, hcpA*	NA	O34:H-
BA3443	*fimA, fimH, lpfA1-2, lpfA2-1, ecpA, hcpA*	NA	O88:H25
BA3836	*fimA, fimH, lpfA1-2, lpfA2-1, ecpA, hcpA*	NA	ONT:H19
BA3148	*fimA, fimH, lpfAO113, lpfA1-2, ecpA, hcpA*	NA	O35:H19
BA956	*fimA, fimH, pilS, pilV, lpfA1-1, ecpA, hcpA*	NA	O111:H15
BA86	*fimA, fimH, lpfA1-2, lpfA2-1, daaC, ecpA*	NA	O76:H19
BA151	*fimA, fimH, lpfA1-2, lpfA2-1, ecpA, hcpA*	NA	ONT:H9
BA3160	*fimA, fimH, lpfA1-2, lpfA2-1, ldaH, ecpA, hcpA*	NA	O110:H-
BA2468	*fimA, fimH, lpfAO113, lpfA1-2, lpfA2-1, ecpA, hcpA*	NA	ONT:H19
BA2775	*fimA, fimH, lpfAO113, lpfA1-2, lpfA2-1, ecpA, hcpA*	NA	O113:H19
BA4013	*fimA, fimH, lpfAO113, lpfA1-2, lpfA2-1, ecpA, hcpA*	NA	O88:H-
BA1738	*fimA, fimH, lpfA1-2, lpfA2-1, ecpA, hcpA*	NA	O80:H26
BA4135	*fimA, fimH, lpfA1-1, lpfA1-2, lpfA2-1, ecpA, hcpA*	NA	O108:H25
BA2923	*fimA, fimH, lpfAO113, lpfA1-2, lpfA1-3, lpfA2-1, ldaH, ecpA, hcpA*	NA	O34:H6

*LA, localized adherence; LAL, localized-adherence like; AA, aggregative adherence; DA, diffuse adherence; UND, undetermined; NA, non-adherent.*

### ECP Is Differentially Produced by aEPEC That Harbor a Functional *ecp* Operon

**FIGURE 2:**
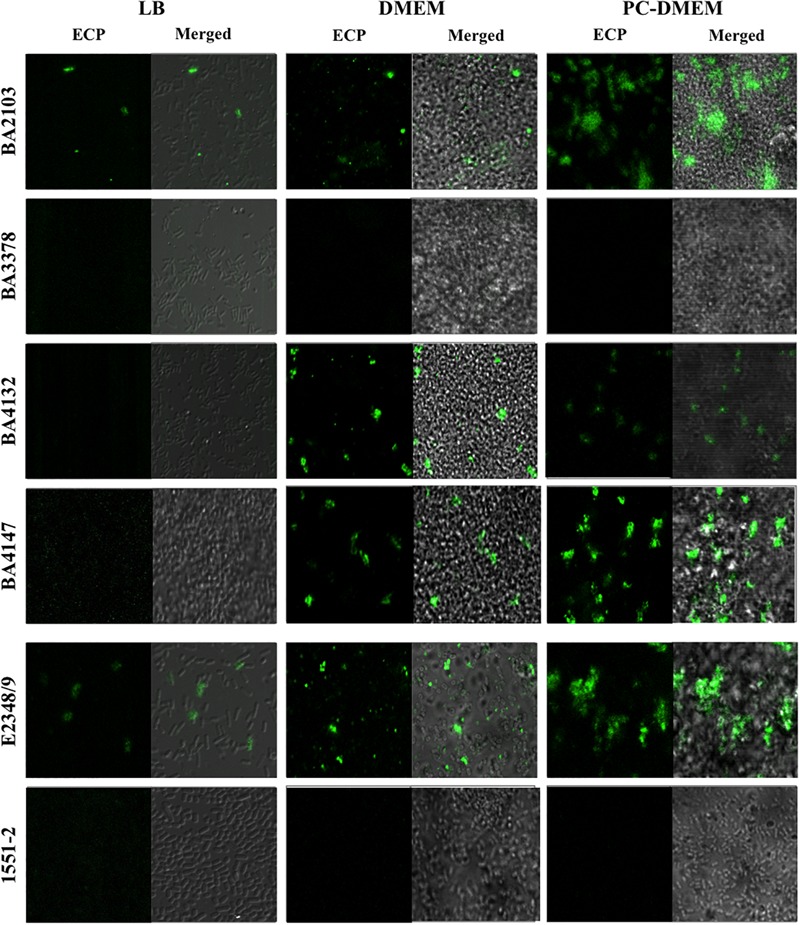
Growth medium-dependent production of ECP visualized by confocal microscopy. aEPEC strains BA2103, BA3378, BA4132, BA4147 grown in LB, DMEM, and PC-DMEM were reacted with rabbit anti-EcpA antibody and goat anti-rabbit IgG antibody conjugated with FITC. E2348/69 and 1551-2 were used as positive and negative controls of ECP production, respectively. Images were originally taken at 1,000× magnification.

**FIGURE 3:**
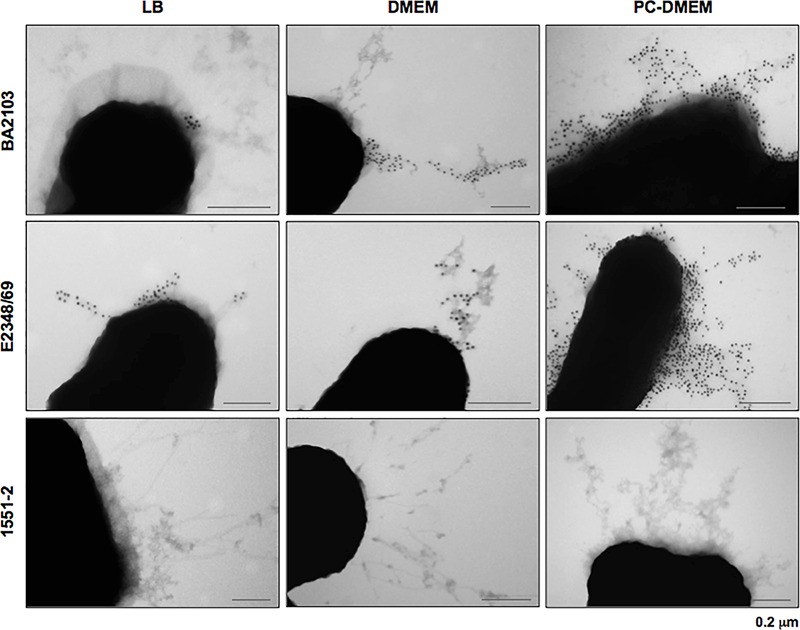
Growth medium-dependent production of ECP visualized by TEM. aEPEC strain BA2103 grown in PC-DMEM, DMEM and LB was reacted with rabbit anti-EcpA antibody and goat anti-rabbit IgG antibody conjugated with 10 nm colloidal gold particles. E2348/69 and 1551-2 were used as positive and negative controls of ECP production, respectively. Bars: 0.2 μm.

The 22 LAL *ecpA+* aEPEC strains contained also the *ecpR, ecpB, ecpC, ecpD*, and *ecpE*, suggesting that the ECP operon is highly conserved among aEPEC (data not shown). To better understand the correlation between the expression of *ecpA* and production of ECP in different culture conditions we selected four representative LAL aEPEC strains (BA2103, BA3378, BA4132, and BA4147), all of which display different fimbrial profiles (**Table 2**). All the six-*ecp* genes were expressed in the four strains regardless of the growth medium (Supplementary Figure S1). However, among the LB-grown bacteria only BA2103 produced ECP. When cultivated in DMEM and PC-DMEM three (BA2103, BA4132, and BA4147) of the four strains produced ECP. Despite the fact that the entire *ecp* operon was expressed in BA3378, this strain did not produce ECP in any of the growth conditions tested (**Figure 2**). In general, a noteworthy increase in ECP production is apparent when the strains were grown in PC-DMEM in comparison to DMEM (**Figure 2**). Confirmation of ECP production by strain BA2103 was demonstrated by TEM and immunogold labeling (**Figure 3**).

## Discussion

tEPEC adherence to enterocytes is multifactorial involving the formation of localized microcolonies on host epithelial cells, which is mediated by BFP, promoting bacterium-cell and bacterium–bacterium interactions (Girón et al., 1991; Tobe and Sasakawa, 2001); followed by the interaction of the intimin adhesin with its receptor Tir triggering the attaching and effacing (A/E) lesion (Jerse et al., 1990; Kenny et al., 1997). On the other hand, the initial interaction of aEPEC with host epithelial cells is instead mediated by distinct cell surface appendages (Hernandes et al., 2006, 2011; Scaletsky et al., 2010; Gomes et al., 2011; Hu and Torres, 2015).

Here, we evaluated a collection of 72 aEPEC for the presence and distribution of several *E. coli* major fimbrial subunit genes known to be involved in bacterial adhesion processes. In agreement with previous reports, none of the genes encoding EAEC fimbriae or the CFs of ETEC were present in the aEPEC strains studied (Vieira et al., 2001; Gomes et al., 2004; Afset et al., 2006). On the other hand, *ecpA, fimA, fimH, hcpA, lpfA_O113_* and polymorphic variants of *lpf* (A1-1, A1-2, A1-3, and A2-1) genes were present in the majority (94–100%) of the strains, while *pilS, pilV, sfpA, daaC, papA*, and *sfa* genes were detected at lower frequency (1–8%) regardless of their adherence pattern. Since each strain studied displayed a specific genetic fimbrial profile, these data indicated that there was no correlation between fimbrial gene profile and adherence pattern phenotypes, as similarly elsewhere described (Afset et al., 2006; Hernandes et al., 2009; Gomes et al., 2016).

The high frequency of *ecpA, hcpA, lpf, fimH*, and *fimA* genes among aEPEC strains indicates that they have likely been conserved to play a role in aEPEC adhesion. It is possible that the acquisition of different gene combinations in specific lineages of *E. coli* may contribute to the emergence of virulent strains (Afset et al., 2006; Bando et al., 2009; Scaletsky et al., 2009). It remains to be determined if recent aEPEC clinical strains are also heterogeneous in terms of their fimbrial adhesins profile. The presence of *papA, sfa, pilS, pilV, ldaH*, and/or *daaC* genes in certain strains deserves further investigation regarding their role as adhesins in host colonization.

Amongst the genes studied, *ecpA* was detected in 100% of our aEPEC collection. The ECP mediates host cell adherence and colonization by both pathogenic and commensal *E. coli* strains (Rendón et al., 2007). While the role of the ECP in adherence to epithelial cells has been demonstrated for several *E. coli* pathotypes, its involvement in the LAL phenotype by aEPEC has not been investigated. The high prevalence of this gene among aEPEC strains and other *E. coli* pathotypes isolated from several studies was previously described (Pouttu et al., 2001; Rendón et al., 2007; Blackburn et al., 2009; Scaletsky et al., 2010). The presence of all the genes responsible for ECP biogenesis in aEPEC has not yet been investigated. Here, we found that all the six-*ecp* genes were present in the 22 LAL-aEPEC strains studied suggesting that the *ecp* operon is highly conserved.

The expression of the *ecpRABCDE* operon and production of ECP was investigated in four aEPEC growing in rich and minimal culture media. The production of virulence markers is still necessary to be evaluated in order to elect an antigen for diagnosis and/or prevention of aEPEC infection. We found that all the strains transcribed the *ecp* genes when cultured in the three growth conditions tested. In terms of ECP production, only BA2103 produced ECP when the strains were grown in LB rich medium. These data are in agreement with previous reports that showed that some ETEC and EHEC strains do not produce ECP in CF antigen agar and LB, respectively (Rendón et al., 2007; Blackburn et al., 2009). Three strains produced ECP when cultivated in DMEM and PC-DMEM. Interestingly, an apparent increase in the number of bacteria producing ECP was noted when the strains were cultured in PC-DMEM in comparison with DMEM, as the reactivity of the anti-EcpA antibody was remarkably more intense in PC-DMEM grown bacteria. This data suggest that eukaryotic cell-derived molecules present in PC-DMEM might trigger expression of the *ecp* operon and synthesis of ECP by aEPEC.

As production of ECP was not detected in aEPEC BA3378 in any growth condition tested, it is possible that this strain produces a variant of EcpA, which is not recognized by the anti-ECP employed, or is under strict regulation. Thus, post-transcriptional factors possibly regulate ECP production in some strains, as in BA3378 of our study. In fact, the operon regulator EcpR/MatA was shown to have dual role in the control of operon expression in a neonatal meningitis *Escherichia coli* (NMEC) strain (IHE 3034) via *mat* promoter, either by a positive autoregulatory circuit or repressing the negative control exerted by H-NS (Lehti et al., 2013). EcpR/MatA is also able to increase the stability of the processed *ecpA* mRNA under some conditions as low growth temperature, acidic pH or elevated levels of acetate (Lehti et al., 2013). The disparity in stability may be due to temperature-dependent conformation of *ecpA* transcript as shown for mRNA of the major cold-shock protein CspA (Giuliodori et al., 2010). Small RNAs (sRNAs) could be another post-transcriptional system involved in ECP production, which generally demands the RNA chaperone Hfq and use different mechanisms, like binding to the ribosome-binding site (RBS) and blocking translation, binding to an anti-RBS hairpin and activating translation, or by the recruitment of RNases destabilizing transcripts (Waters and Storz, 2009). Also, Gruber and Sperandio (2015) recently reported that in EHEC, GlmY and GlmZ selectively destabilize parts of the *LEE4* and *LEE5* transcripts playing an important role in the post-transcriptional regulation of A/E lesion formation. Genes within the GlmY and GlmZ regulon include stress-related genes, virulence factors, genes involved in osmoregulation and adhesins, which is the case of ECP. The role of these post-transcriptional regulators in ECP production by our strains is currently under investigation by our group.

The reactivity of anti-EcpA antibody was remarkably more intense in bacterial cell grown in PC-DMEM than in DMEM. Similar to tEPEC strains, in which ECP and BFP act in combination to promote adherence, it is reasonable to suggest that ECP could act together with other adhesins of aEPEC to favor gut colonization and survival in and outside the host. Also, the high prevalence of ECP among aEPEC indicates that this adherence factor has been maintained during evolution to ensure intestinal colonization.

In summary, our data demonstrated that different *E. coli* fimbrial genes profiles are present in the aEPEC strains studied and that there is no correlation between these profiles and adherence patterns. The high prevalence of ECP, HCP, and type 1 fimbriae among aEPEC suggest that they are likely to contribute to cell adhesion. We also demonstrated that culture conditions influenced ECP production by aEPEC strains, likely attributed to post-transcriptional regulation.

## Author Contributions

DM, JN, KN, BY, FV, YM-L, FM, CA, WE, and RP: conception and design. DM, JN, NF, CM, KN, BY, FV, FM, and CA: acquisition and analysis. DM, JN, NF, CM, KN, BY, FV, YM-L, JG, FM, CA, WE, and RP: interpretation and substantial contributions. DM, CM, YM-L, JG, FM, CA, WE, and RP: drafting the work or revising it critically. DM, JG, CA, WE, and RP: final approval of the version to be published.

## Conflict of Interest Statement

The authors declare that the research was conducted in the absence of any commercial or financial relationships that could be construed as a potential conflict of interest.
