# Supplementary material for: Distribution of Major Pilin Subunit Genes Among Atypical Enteropathogenic Escherichia coli and Influence of Growth Media on Expression of the ecp Operon
Source: Front Microbiol. 2018 May 15;9:942. doi: 10.3389/fmicb.2018.00942 (PMC5962669; doi:10.3389/fmicb.2018.00942)
Supplement: Supplementary file 1 [file Table_1.pdf]

Supplementary Table S1. Control strains, primers sequences and PCR conditions to detect fimbriae-encoding genes of *E. coli*

Gene	Sequences (5´- 3´)	Annealing temperature / Incubation	Fragment (pb)	Primers reference	Control strains	Strain reference
fimA	(F) CTGTCGGCTCTGTCCCTCAGT<br>(R) GATGCGGTACGAACCTGTCCTAA	65ºC / 1 min	161	Nowrouzian et al., 2005	J96 (+)<br>UEL13 (–)	Hull et al., 1981<br>Rocha et al., 2007
fimH	(F) GACGTCACCTGCCCTCCGGTA<br>(R) TGCAGAACGGATAAGCCGTGG	63ºC / 1 min	508	Hernandes et al., 2011	J96 (+)<br>UEL13 (–)	Hull et al., 1981<br>Rocha et al., 2007
papA	(F) CACATTATCACCATCTTTC<br>(R) TCTATTGATTTTGGACAGC	50ºC / 1 min	306	This study<br>(GenBank: NC_007946)	J96 (+)<br>UEL13 (–)	Hull et al., 1981<br>Rocha et al., 2007
sfaD-E	(F) CTCCGGAGAACTGGGTGCATCTTAC<br>(R) CGGAGGAGTAATTACAAACCTGGCA	50ºC / 40 sec	410	Le Bouguénec et al., 1992	RS218 (+)<br>DH5α (–)	Johnson et al., 2001<br>Sambrook et al., 1989
bfpA	(F) GGTCTGTCTTTGATTGAATC<br>(R) TTTACATGCAGTTGCCGCTT	55ºC / 1 min	485	This study<br>(GenBank: NC_002142)	E2348/69 (+)<br>C600 (–)	Levine et al., 1985<br>Sambrook et al., 1989
ecpA	(F) GCCGCTGATGATGGAGAAAG<br>(R) GCAACAGCCAAAAAAGACACC	56ºC / 1 min	384	Saldaña et al., 2009	E2348/69 (+)<br>1551-2 (-)	Levine et al., 1985<br>Yamamoto et al., 2017
ldaH	(F) CCCCGGTTTTACGCCTTTTGT<br>(R) CGTAACCCTGCCGTCCGATAGA	64ºC / 1 min	391	This study<br>(GenBank: AY858803.1)	*E. coli 22* (+)<br>C600 (–)	Scaletsky et al., 2005<br>Sambrook et al., 1989

aggA	(F) GCGTTAGAAAGACCTCCAATA	55ºC / 1 min	462	Bernier et al., 2002	17-2 (+)	Nataro et al., 1993
	(R) GCCGGATCCTTAAAAATTAATTCCGGC				C600 (–)	Sambrook et al., 1989
aafA	(F) ACATGCATGCAAAAAATCAGAATGTTTGTT	63ºC / 1 min	550	Czeczulin et al, 1997	042 (+)	Vial et al., 1988
	(R) CGGGATCCATTTGTCACAAGCTCAGC				C600 (–)	Sambrook et al., 1989
agg3A	(F) GTATCATTGCGAGTCTGGTATTCAG	60ºC / 1 min	462	Bernier et al., 2002	RN785-1 (+)	Zamboni et al., 2004
	(R) GGGCTGTTATAGAGTAACTTCCAG				C600 (–)	Sambrook et al., 1989
agg4A	(F) TGAGTTGTGGGGCTAYCTGGA	57ºC / 1 min	169	Boisen et al., 2008	BA1116 (+)	Abe et al., 2009
	(R) CACCATAAGCCGCCAAATAAGC				DH5α (–)	Sambrook et al., 1989
pilS	(F) ATGAGCGTCATAACCTGTTC	50ºC / 1 min	534	Dudley et al., 2006	C1096 (+)	Cobeljic et al., 1996
	(R) CTGTTGGTTTCCAGTTTGAT				C600 (–)	Sambrook et al., 1989
pilV	(F) ATGCAAAAAGACAACGATAA	50ºC / 1 min	1194	Dudley et al., 2006	C1096 (+)	Cobeljic et al., 1996
	(R) TTAATTGAGCGTTACACACG				C600 (–)	Sambrook et al., 1989
lngA	(F) AAAAATCGCCAAATACCAT	55ºC / 45 sec	588	This study	E9034A (+)	Girón et al., 1994
	(R) GTTTGTCCATTGTTACCT			(GenBank: AF004308)	C600 (–)	Sambrook et al., 1989
cfaB	(F) GCTCTGACCACAATGTTTG	54ºC / 1 min	364	Ghosal et al., 2007	H10407 (+)	Evans et al., 1975
	(R) TTACACCGGATGCAGAATA				C600 (–)	Sambrook et al., 1989

cooA	(F) TTGACCTTCTGCAATCTGA	54ºC / 1 min	324	Ghosal et al., 2007	170A1 (+)	This study
	(R) CATCTGCATGGATTGTTGAAAG				C600 (–)	Sambrook et al., 1989
cotA	(F) GAGAAAAATATCACTGTAACTG	57ºC / 1 min	385	This study	E4833 (+)	Guth et al., 1994
	(R) TATTAGTTTGCTGGGTGCTTC			(GenBank: Z47800.1)	DH5α (–)	Sambrook et al., 1989
cstA	(F) GGTGGGTGTTTTGACTCTT	54ºC / 1 min	264	Ghosal et al., 2007	PB176 (+)	Evans and Evans, 1978
	(R) TGTTCGTTACCTTCAGTGG				C600 (–)	Sambrook et al., 1989
c*ofA*	(F) GCCTTCTGGAAGTCATCAT	52ºC / 40 sec	437	Ghosal et al., 2007	220A1 (+)	This study
	(R) TGCCACATACTCCCAGTTA				DH5α (–)	Sambrook et al., 1989
csaA	(F) TTTTGCAAGCTGATGGTAG	54ºC / 1 min	250	Ghosal et al., 2007	E8775 (+)	Thomas et al., 1982
	(R) TCTGCAGGTTCAAAAGTCA				C600 (–)	Sambrook et al., 1989
csfA	(F) CGGATTGGATATACCGTTT	54ºC / 1 min	453	Ghosal et al., 2007	4961-2 (+)	Giraldi and Guth, 1993
	(R) TCAACAGCAAATGTTACCG				C600 (–)	Sambrook et al., 1989
cssA	(F) TTTTGCAAGCTGATGGTAG	54ºC / 1 min	250	Ghosal et al., 2007	E17018A (+)	McConnell et al., 1988
	(R) TCTGCAGGTTCAAAAGTCA				C600 (–)	Sambrook et al., 1989
daaC	(F) GTTCTGACGCACCTCTATCCG	64ºC / 1 min	286	This study	C1845 (+)	Bilge et al., 1989
	(R) CATTGGACCCTGGCGTGTTAG			(GenBank: EU010379.1)	DH5α (–)	Sambrook et al., 1989

sfpA	(F) AGCCAAGGCCAAGGGATTATTA<br>(R) TTAGCAACAGCAGTGAAGTCTC	64ºC - 1 min	440	Brunder et al., 2001	O157:H⁻ (+)<br>C600 (–)	Blanco et al., 1995<br>Sambrook et al., 1989
hcpA	(F) TCGCTAGTTGCTGACAGATTT<br>(R) AATGTCTGTTGTGTGCGACTG	50ºC / 1 min	868	Xicohtencatl-Cortes et al., 2007	EDL933 (+)<br>H10407 (-)	Perna et al., 2001<br>Evans et al., 1975
lpfA $_{O113}$	(F) ATGAAGCGTAATATTATAG<br>(R) TTATTTCTTATATTCGAC	52ºC / 1 min	573	Doudhty et al., 2002	STEC50 (+)<br>DH5α (–)	Vaz et al., 2006<br>Sambrook et al., 1989
lpfA1-1	(F) GTGCTGGATTCACCACTATTCATCG<br>(R) AGTTGGTGATAAATCACCAT	59ºC / 30 sec	222	Torres et al., 2009	E2348/69(+)<br>C600 (–)	Levine et al., 1985<br>Sambrook et al., 1989
lpfA1-2	(F) AAGTCTGTATTTACTGCTATG<br>(R) GAAATACAGAACGGTCTGA	57ºC / 30 sec	273	Torres et al., 2009	FV10094 (+)<br>C600 (–)	Torres et al., 2009<br>Sambrook et al., 1989
lpfA1-3	(F) GGTTGGTGACAAATCCCCG<br>(R) CGTCTGGCCTTTACTCAGA	62ºC / 30 sec	244	Torres et al., 2009	EDL933 (+)<br>C600 (–)	Perna et al., 2001<br>Sambrook et al., 1989
lpfA1-5	(F) GGTTGGTGACAAATCCCCG<br>(R) GAGAACCGTCTGGCCTGTTT	60ºC / 30 sec	273	Torres et al., 2009	FV10106 (+)<br>DH5α (–)	Torres et al., 2009<br>Sambrook et al., 1989
lpfA2-1	(F) GGTAGTCTGGCGTCGCCACAGA<br>(R) AATACGAATACCAACGCCG	60ºC / 30 sec	207	Torres et al., 2009	FV10132 (+)<br>C600 (–)	Torres et al., 2009<br>Sambrook et al., 1989

References cited in Supplementary Table S1

Abe, C.M., Trabulsi, L.R., Blanco, J., Blanco, M., Dahbi, G., Blanco, J.E., et al. (2009). Virulence features of atypical enteropathogenic *Escherichia coli* identified by the *eae*(+) EAF-negative stx(-) genetic profile. *Diagn Microbiol Infect Dis*. 64, 357-65.

Bilge, S.S., Clausen, C.R., Lau, W., and Moseley, S.L. (1989). Molecular characterization of a fimbrial adhesin, F1845, mediating diffuse adherence of diarrhea-associated *Escherichia coli* to HEp-2 cells. *J Bacteriol*.171, 4281-9.

Bernier, C., Gounon, P., and Le Bouguénec, C. (2002). Identification of an aggregative adhesion fimbria (AAF) type III-encoding operon in enteroaggregative *Escherichia coli* as a sensitive probe for detecting the AAF-encoding operon family. *Infect Immun*. 70, 4302-11.

Blanco, J.E., Blanco, M., and Blanco, J. (1995). Enterotoxigenic, verotoxigenic, and necrotoxigenic *Escherichia coli* in food and clinical samples. Role of animals as reservoirs of strains pathogenic for humans. *Microbiologia*. 11, 97-110.

Boisen, N., Struve, C., Scheutz, F., Krogfelt, K.A., and Nataro, J.P. (2008). New adhesin of enteroaggregative *Escherichia coli* related to the Afa/Dr/AAF family. *Infect Immun*. 76, 3281-92.

Brunder, W., Khan, A.S., Hacker, J., and Karch, H. (2001). Novel type of fimbriae encoded by the large plasmid of sorbitol-fermenting enterohemorrhagic *Escherichia coli* O157:H(-). *Infect Immun*. 69, 4447-57.

Cobeljić, M., Miljković-Selimović, B., Paunović-Todosijević, D., Velicković, Z., Lepsanović, Z., Zec, N., et al. (1996). Enteroaggregative *Escherichia coli* associated with an outbreak of diarrhoea in a neonatal nursery ward. *Epidemiol. Infect.* 117, 11-16.

Czeczulin, J.R., Balepur, S., Hicks, S., Phillips, A., Hall, R., Kothary, M.H., et al. (1997). Aggregative adherence fimbria II, a second fimbrial antigen mediating aggregative adherence in enteroaggregative *Escherichia coli*. *Infect Immun.* 65, 4135-45.

Doughty, S., Sloan, J., Bennett-Wood, V., Robertson, M., Robins-Browne, R.M., and Hartland, E.L. (2002). Identification of a novel fimbrial gene cluster related to long polar fimbriae in locus of enterocyte effacement-negative strains of enterohemorrhagic *Escherichia coli*. *Infect Immun*. 70, 6761-9.

Dudley, E.G., Abe, C., Ghigo, J.M., Latour-Lambert, P., Hormazabal, J.C., and Nataro, J.P. (2006). An IncI1 plasmid contributes to the adherence of the atypical enteroaggregative *Escherichia coli* strain C1096 to cultured cells and abiotic surfaces. *Infect Immun*. 74, 2102-14.

Evans, D.G., and Evans, D.J. (1978). New surface-associated heat-labile colonization factor antigen (CFA/II) produced by enterotoxigenic of *Escherichia coli* of serogroups O6 and O8. *Infect. Immun.* 21, 638-647.

Evans, D.G., Silver, R.P., Evans, D.J. Jr., Chase, D.G., and Gorbach, S.L. (1975). Plasmid-controlled colonization factor associated with virulence in *Esherichia coli* enterotoxigenic for humans. *Infect Immun*. 12, 656-67.

Ghosal, A., Bhowmick, R., Nandy, R.K., Ramamurthy, T., and Chatterjee, N.S. (2007). PCR-based identification of common colonization factor antigens of enterotoxigenic *Escherichia coli. J Clin Microbiol.* 45, 3068-71.

Giraldi, R., and Guth, B.E. (1993). Presence of colonization factor antigen IV (CS5CS6) in O29:H21 enterotoxigenic *Escherichia coli* isolated from children with diarrhea in Brazil. *Braz J Med Biol Res*. 26, 1251-9.

Girón, J.A., Levine, M.M., and Kaper, J.B. (1994). Longus: a long pilus ultrastructure produced by human enterotoxigenic *Escherichia coli. Mol Microbiol.* 12, 71-82.

Guth, B.E., Aguiar, E.G., Griffin, P.M., Ramos, S.R., and Gomes, T.A. (1994). Prevalence of colonization factor antigens (CFAs) and adherence to HeLa cells in enterotoxigenic *Escherichia coli* isolated from feces of children in São Paulo. *Microbiol Immunol*. 38, 695-701.

Hernandes, R.T., Velsko, I., Sampaio, S.C., Elias, W.P., Robins-Browne, R.M., Gomes, T.A., et al. (2011). Fimbrial adhesins produced by atypical enteropathogenic *Escherichia coli* strains. *Appl Environ Microbiol*. 77, 8391-9.

Hull, R.A., Gill, R.E., Hsu, P., Minshew, B.H., and Falkow, S. (1981). Construction and expression of recombinant plasmids encoding type 1 or D-mannose-resistant pili from a urinary tract infection *Escherichia coli* isolate. *Infect Immun.* 33, 933-8.

Johnson, J.R., Weissman, S.J., Stell, A.L., Trintchina, E., Dykhuizen, D.E., and Sokurenko, E.V. (2001). Clonal and pathotypic analysis of archetypal *Escherichia coli* cystitis isolate NU14. *J Infect Dis*. 184, 1556-65.

Le Bouguénec, C., Archambaud, M., and Labigne, A. (1992). Rapid and specific detection of the *pap*, *afa*, and *sfa* adhesin-encoding operons in uropathogenic *Escherichia coli* strains by polymerase chain reaction. *J Clin Microbiol*. 30, 1189-93.

Levine, M.M., Nataro, J.P., Karch, H., Baldini, M.M., Kaper, J.B., Black, R.E., et al. (1985). The diarrheal response of humans to some classic serotypes of enteropathogenic *Escherichia coli* is dependent on a plasmid encoding an enteroadhesiveness factor. *Infect Dis.* 152, 550-9.

McConnell, M.M., Thomas, L.V., Willshaw, G.A., Smith, H.R., and Rowe, B. (1988). Genetic control and properties of coli surface antigens of colonization factor antigen IV (PCF8775) of enterotoxigenic *Escherichia coli. Infect Immun.* 56, 1974-80.

Nataro, J.P., Yikang, D., Giron, J.A., Savarino, S.J., Kothary, M.H., and Hall, R. (1993). Aggregative adherence fimbria I expression in enteroaggregative *Escherichia coli* requires two unlinked plasmid regions. *Infect Immun.* 61, 1126-31.

Nowrouzian, F.L., Monstein, H.J., Wold, A.E., and Adlerberth, I. (2005). Effect of human milk on type 1 and P-fimbrial mRNA expression in intestinal *Escherichia coli* strains. *Lett Appl Microbiol* 40, 74-80.

Perna, N.T., Plunkett, G.R.D., Burland, V.; Mau, B.; Glasner, J. D.; Rose, D. J., et al. (2001). Genome sequence of enterohaemorrhagic *Escherichia coli* O157:H7. Nature. 409, 529-533.

Rocha, S.P., Elias, W.P., Cianciarullo, A.M., Menezes, M.A., Nara, J.M., Piazza, R.M., et al. (2007). Aggregative adherence of uropathogenic *Proteus mirabilis* to cultured epithelial cells. *FEMS Immunol Med Microbiol.* 51, 319-26.

Saldaña, Z., Erdem, A.L., Schüller, S., Okeke, I.N., Lucas, M., Sivananthan, A., et al. (2009). The *Escherichia coli* common pilus and the bundle-forming pilus act in concert during the formation of localized adherence by enteropathogenic *E. coli*. *J Bacteriol*. 191, 3451-61.

Sambrook, J., Fritsch, E.F., and Maniatis, T. (1989). *Molecular cloning: a Laboratory Manual*. New York: Cold Spring Harbor Laboratory Press.

Scaletsky, IC., Michalski, J., Torres, A.G., Dulguer, M.V., and Kaper, J.B. (2005). Identification and characterization of the locus for diffuse adherence, which encodes a novel afimbrial adhesin found in atypical enteropathogenic *Escherichia coli*. *Infect Immun*. 73, 4753-65.

Thomas, L.V., Cravioto, A., Scotland, S. M., and Rowe, B. (1982). New fimbrial antigenic type (E8775) that may represent a colonization factor in enterotoxigenic *Escherichia coli* in humans. *Infect. Immun*. 35, 1119- 1124,

Torres, A.G., Blanco, M., Valenzuela, P., Slater, T.M., Patel, S.D., Dahbi, G., et al. (2009). Genes related to long polar fimbriae of pathogenic *Escherichia coli* strains as reliable markers to identify virulent isolates. *J Clin Microbiol*. 47, 2442-51.

Vaz, T.M., Irino, K., Nishimura, L.S., Cergole-Novella, M.C., and Guth, B.E. (2006). Genetic heterogeneity of Shiga toxin-producing

Escherichia coli strains isolated in Sao Paulo, Brazil, from 1976 through 2003, as revealed by pulsed- field gel electrophoresis. *J. Clin. Microbiol.* 44, 798-804.

Vial, P.A., Robins-Browne, R., Lior, H., Prado, V., Kaper, J.B., Nataro, J.P., et al. (1988). Characterization of enteroadherent-aggregative *Escherichia coli*, a putative agent of diarrheal disease. *J. Infect. Dis*. 158, 70-79.

Xicohtencatl-Cortes, J., Monteiro-Neto, V., Ledesma, M.A., Jordan, D.M., Francetic, O., Kaper, J.B., et al. (2007). Intestinal adherence associated with type IV pili of enterohemorrhagic *Escherichia coli* O157:H7. *J Clin Invest*. 117, 3519-29.

Yamamoto, D., Hernandes, R.T., Liberatore, A.M., Abe, C.M., Souza, R.B., Romão, F.T., et al. (2017). *Escherichia albertii*, a novel human enteropathogen, colonizes rat enterocytes and translocates to extra-intestinal sites. *PLoS One.* 12: 2 doi: 10.1371/journal.pone.0171385.

Zamboni, A., Fabbricotti, S.H., Fagundes-Neto, U., and Scaletsky, I.C. (2004). Enteroaggregative *Escherichia coli* virulence factors are found to be associated with infantile diarrhea in Brazil. *J. Clin. Microbiol*. 42, 1058- 1063.
