# Supplementary material for: Distribution of Major Pilin Subunit Genes Among Atypical Enteropathogenic Escherichia coli and Influence of Growth Media on Expression of the ecp Operon
Source: Front Microbiol. 2018 May 15;9:942. doi: 10.3389/fmicb.2018.00942 (PMC5962669; doi:10.3389/fmicb.2018.00942)
Supplement: Supplementary file 2 [file Image_1.PDF]

Supplementary Figure 1

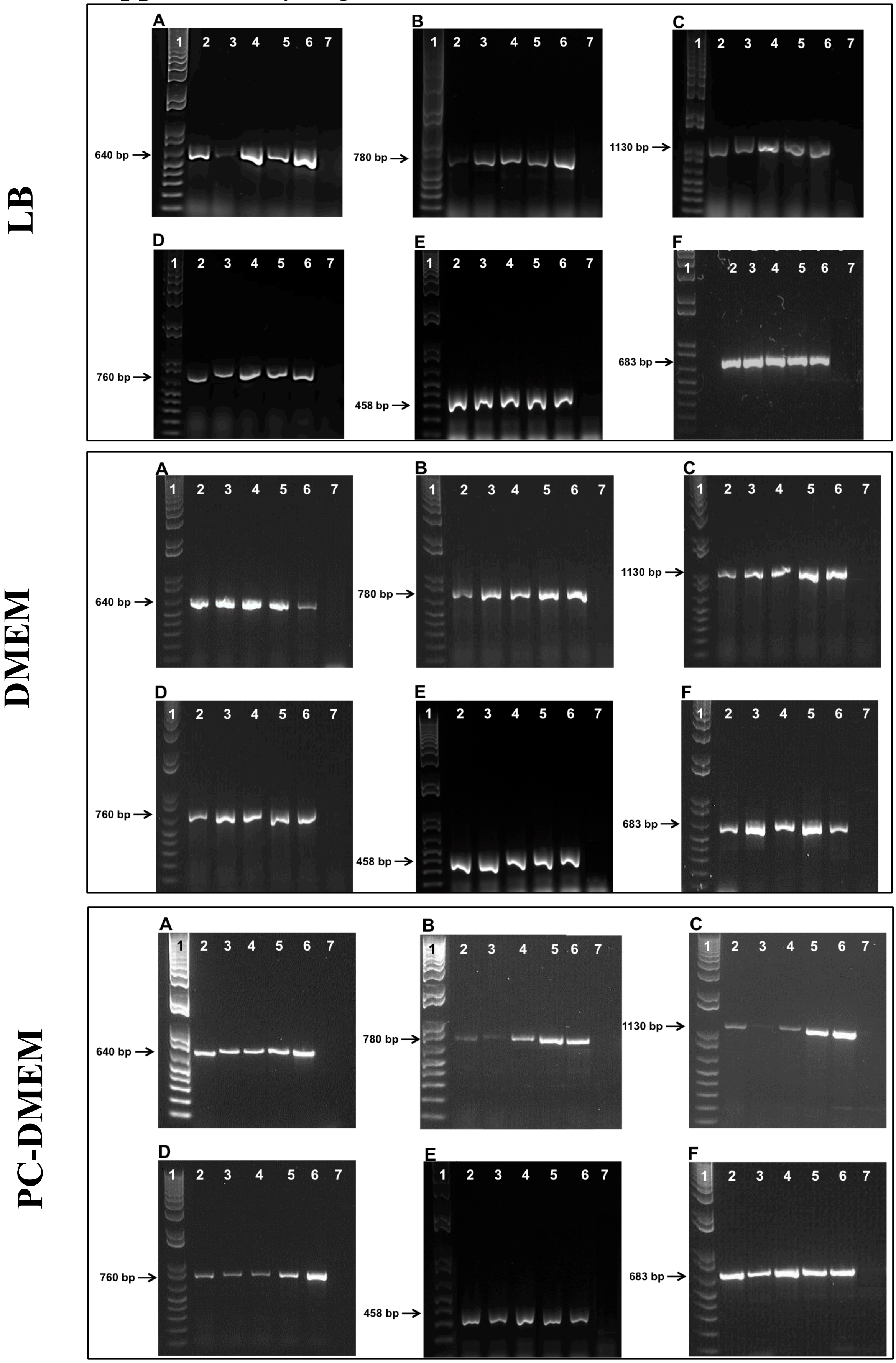

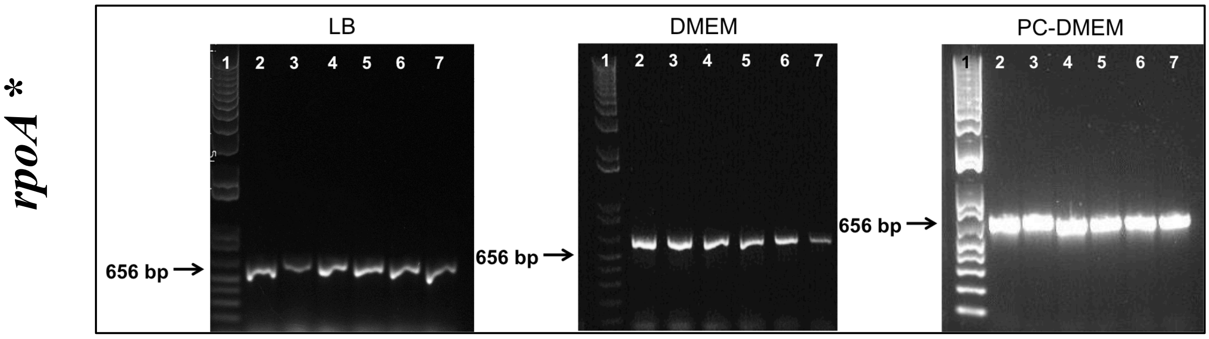

Figure S1. DNA profile of *ecp* operon by RT-PCR of strains grown in LB, DMEM and PC-DMEM. A. *ecpA;* **B.** *ecpB;* **C.** *ecpC;* **D.** *ecpD;* **E.** *ecpE;* **F.** *ecpR.* **1.** DNA Ladder (1 Kb Plus); **2.** BA2103; **3.** BA3378; **4.** BA4132; **5.** BA4147; **6.** E2348/69; **7.** 1551-2. The expected product size is indicated by the arrow of each gel *The last set of agarose gels show *rpoA* amplification as control of RNA extractions.
